# The self-management abilities test (SMAT): a tool to identify the self-management abilities of adults with bronchiectasis

**DOI:** 10.1038/s41533-021-00265-5

**Published:** 2022-01-14

**Authors:** Katelyn R. Smalley, Lisa Aufegger, Kelsey Flott, Erik K. Mayer, Ara Darzi

**Affiliations:** 1 NIHR Imperial PSTRC (Patient Safety and Translational Research Centre), Institute of Global Health Innovation, Department of Surgery & Cancer, Imperial College London, London, UK; 2 Community and Primary Care Research Group, Plymouth Institute of Health and Care Research, University of Plymouth, Plymouth, UK

**Keywords:** Patient education, Respiratory tract diseases, Health services

## Abstract

Bronchiectasis is an increasingly common chronic respiratory disease which requires a high level of patient engagement in self-management. Whilst the need for self-management has been recognised, the knowledge and skills needed to do so— and the extent to which patients possess these—has not been well-specified. On one hand, understanding the gaps in people’s knowledge and skills can enable better targeting of self-management supports. On the other, clarity about what they *do* know can increase patients’ confidence to self-manage. This study aims to develop an assessment of patients’ ability to self-manage effectively, through a consensus-building process with patients, clinicians and policymakers. The study employs a modified, online three-round Delphi to solicit the opinions of patients, clinicians, and policymakers (*N* = 30) with experience of bronchiectasis. The first round seeks consensus on the content domains for an assessment of bronchiectasis self-management ability. Subsequent rounds propose and refine multiple-choice assessment items to address the agreed domains. A group of ten clinicians, ten patients and ten policymakers provide both qualitative and quantitative feedback. Consensus is determined using content validity ratios. Qualitative feedback is analysed using the summative content analysis method. Overarching domains are *General Health Knowledge, Bronchiectasis-Specific Knowledge, Symptom Management, Communication*, and *Addressing Deterioration*, each with two sub-domains. A final assessment tool of 20 items contains two items addressing each sub-domain. This study establishes that there is broad consensus about the knowledge and skills required to self-manage bronchiectasis effectively, across stakeholder groups. The output of the study is an assessment tool that can be used by patients and their healthcare providers to guide the provision of self-management education, opportunities, and support.

## Introduction

Self-management is critical in chronic diseases like bronchiectasis, since much of the treatment takes place at home, outside the supervision of healthcare professionals. Many patients over time become ‘experts’ with respect to their condition, but that expertise has been contested and not rigorously specified^1,2^. Policymakers have long recognised that patients have a role to play in managing chronic illnesses, through self-care, communication with healthcare providers and moderating the frequency and intensity of the care they receive^3–5^. However, clarity is lacking with respect to what patients can do (and how that varies by individual), what they must be able to do (for safety reasons) and what they should be responsible for (as a non-healthcare professional contributing to the management of their chronic disease). Others have highlighted that some degree of variation on these dimensions is appropriate; thus it is necessary to tailor self-management interventions to individual patients^6^. This is particularly critical in bronchiectasis, for which treatment is variable and personalised self-management plans are recommended for all^7^. This study explored what patients must know and be able to do to safely self-manage bronchiectasis. In consultation with a multi-stakeholder expert panel, we developed an assessment tool to identify the extent to which adults with bronchiectasis have those necessary knowledge and skills. Bronchiectasis is a chronic respiratory disease that is characterised by symptoms such as dyspnoea, productive cough, chest discomfort and recurrent chest infections^8^. It is an increasingly common condition, with an estimated prevalence of 212,000 people in the UK, and a 20% increase in prevalence from 2008 to 2012^9^. An estimated 500 per 100,000 people in the UK have been diagnosed with bronchiectasis, which equates to 50 patients in a GP practice of 10,000^10^. A diagnosis of bronchiectasis is confirmed radiologically by permanent, abnormal dilation of the bronchi^11^. Bronchiectasis can result from diverse causes, and often the cause is unknown^12^. The disease is characterised by recurrent infections called exacerbations, and the goals of treatment are to prevent exacerbations, halt disease progression and minimise symptoms^11^. Bronchiectasis is often termed a ‘vicious circle of infection and inflammation’ (British Thoracic Society^8^). The cyclical nature of the disease provides an opportunity to learn over time. The daily process of managing symptoms, maintaining exercise and medication regimens, and recognising signs of exacerbation or deterioration, provide people with bronchiectasis ample opportunities to engage in self-management. Self-management can be challenging to characterise because it can mean different things to different people in different contexts^13^. Most commonly, the term self-management can be used to describe both the daily activities that patients undertake to maintain control over their disease, but also interventions that train patients in these activities^14^. Self-management can refer to a wide range of behaviours, including exercise, symptom monitoring and asking follow-up questions in healthcare appointments^15^. Some self-management activities (e.g. routine airway clearance) are disease-specific, and others (e.g. smoking cessation) are universal. Behaviour change interventions called self-management programmes have become commonplace in a variety of chronic conditions, but there is a paucity of data about the optimal content of such programmes tailored to bronchiectasis. A recent systematic review found only two bronchiectasis self-management programmes globally^16^. These studies, both from the UK, were unable to establish benefits on their primary outcome of health-related quality of life and did not report on some clinically-relevant outcomes like exacerbations requiring antibiotics^16^. On the other hand, qualitative research suggests that information deficits may be a barrier to effective self-management of bronchiectasis^17^. Both knowledge, defined as ‘awareness of the existence of something’, and skills, defined as ‘ability or proficiency acquired through practice’^18^, are critical capabilities for self-management. We term the knowledge and skills needed to self-manage a chronic disease self-management ability. In this study, we work with an expert panel of clinicians, policymakers and people with bronchiectasis to define the knowledge and skills needed to self-manage bronchiectasis safely and to develop an assessment tool to measure the extent to which patients possess those knowledge and skills. In accordance with best practices for assessment development, the literature was searched for potentially relevant assessments that would supersede the need for a new tool^19^. Four generic self-management-related assessments were identified: the Patient Activation Measure^20^, the Chronic Disease Self-Efficacy Scale^21^, the Test of Functional Health Literacy in Adults^22^ and the Health Education Impact Questionnaire^23^. Aside from being unable to address the specific information needs of patients with bronchiectasis as identified by Hester et al. (2018), they also assessed concepts other than self-management knowledge and skills (namely, patient activation and self-efficacy)^17^. Two respiratory-specific assessments were identified. The Bristol COPD Knowledge Questionnaire was the only knowledge assessment identified^24^. As suggested by the name, this questionnaire assessed knowledge only, and not skills. Further, whilst there is considerable overlap in the knowledge and skills needed to manage COPD and bronchiectasis, there are important differences that must also be taken into account. Finally, the Lung Information Needs Questionnaire assessed patients’ perception of how well clinicians communicated information to them^25^. These perceptions are distinct from self-management ability per se, which entails being able to reproduce or act on that information. We did not identify any assessments that measure patients’ self-management knowledge and skills directly. Since these are two key outcomes of self-management interventions, this is a significant gap in the literature. This is especially true for bronchiectasis, for which disease-related information is particularly important^17^. For all of these reasons, the existing literature supported the development of a new assessment tool that would measure self-management ability directly, and specifically with respect to bronchiectasis. The aim of this work was to co-develop a measure of patients’ ability to self-manage bronchiectasis, with patients, clinicians, and policymakers with experience of the disease. Two specific objectives serve to meet this aim:

- To achieve a consensus definition of effective self-management in terms of knowledge and skills.
- To produce a measurement tool of patients’ ability to self-manage bronchiectasis.

## Methods

### Study design

This study employed an online, modified Delphi method^26^^,^^27^. The Delphi technique is an anonymous, iterative process to solicit expert opinion through a series of structured questionnaires, with the purpose to either gain consensus or identify systematic disagreement^28^.

Delphi is a flexible method that has been used for multiple purposes in health research, including assessment development^29,30^. It has the advantage of collecting expert opinions asynchronously, allowing the input of multiple individuals without geographic or scheduling limitations^31^. For the purposes of this study, it also allowed the participation of seriously chronically ill individuals who could complete the questionnaires from home. The anonymity of Delphi participants reduces the risk of power dynamics confounding responses^32,33^. This study modified the method to first achieve consensus on content domains, and then further develop assessment items that address those domains.

### Participation

This study, which took place in the UK, solicited expert opinions from three stakeholder groups:

1. Adult patients with bronchiectasis (either with or without cystic fibrosis).
2. Clinicians (general practitioners, respiratory consultants/specialists, respiratory physiotherapists, specialist nurses, pharmacists) who currently work in a clinical capacity, and who have experience treating bronchiectasis.
3. Policymakers with experience of bronchiectasis (broadly conceived as people whose current responsibilities include not only direct patient care, but also the development of policies and procedures for the care of these patients either at the multi-disciplinary team, trust, regional or national level).

This range of stakeholders were chosen to provide a multi-faceted view of self-management ability. Reflecting sample size guidance for Delphi studies, ten participants represented each stakeholder group, for a panel of 30 participants in total^34^.

### Recruitment and study setting

Patients were recruited online via the Voice Global and People in Research networks of lay researchers, Twitter, the British Lung Foundation’s Breathe Easy support groups, and through informal networks of lay representatives at Imperial College. Clinicians were recruited via respiratory professional societies such as BronchUK and the British Thoracic Society, publicly available profiles and personal references. Policymakers were identified via public research and policy profiles, and leadership of professional societies, research groups or major clinical studies of bronchiectasis. The panel was recruited from across the UK and provided written informed consent to participate.

The Delphi was conducted in three rounds, over the course of 9 months. The contents of the Delphi questionnaires were derived from the literature on self-management of bronchiectasis and consultation with respiratory clinicians and patient experts. The questionnaires were administered online via Qualtrics software licensed to Imperial College London. Data were stored and managed in Excel, and quantitative analyses were completed either in R or Excel. Data were stored on Imperial’s Big Data Analytic Unit (BDAU) secure server in accordance with UK Health Research Authority (HRA) ethical approval (IRAS # 250224).

### Data collection

The purpose of Round 1 was to agree to the content domains that should be covered by an assessment of self-management ability for bronchiectasis. A preliminary list of content domains was generated through interviews with stakeholders and a literature review. The content of self-management programmes for bronchiectasis and other respiratory diseases^16,35,36^, taxonomies of behaviour change and self-management support^37,38^ and other assessments of preparedness for self-management^20,21,24,25^ were consulted in the development of this list.

Semi-structured interviews with respiratory patients, clinicians and policymakers (who were not involved in the present study) provided insights on the key knowledge, skills and attitudes needed to effectively self-manage long-term respiratory diseases (results not yet published).

Participants were presented with items that may be related to a patient’s ability to self-manage bronchiectasis effectively. They were asked to rank how important they believed these items were to a patient’s ability to self-manage this disease on a seven-point Likert Scale, from 1 = not relevant to 7 = essential^19^. Questions were posed in the format: ‘To what extent do you believe it is important for patients to…?’ Participants were provided with space to explain their answers.

The purpose of Round 2 was to solicit opinions on potential assessment items addressing the content domains agreed in Round 1. Round 2 was divided into two sections—Round 2a and Round 2b. Round 2a included items for which consensus was achieved in Round 1. For these items, participants were asked to react to a draft assessment question that might be proposed to patients to test the domain. Assessment questions took the form of simple multiple choice, True/False, vignettes, and ‘check all that apply’. Round 2b focussed on items for which consensus was not achieved in the previous round. Participants were asked to reconsider the item in light of the responses of other participants, and either maintain or change their opinion on the inclusion of the item.

Finally, in Round 3, participants reviewed the revised assessment, confirmed the items to be included, and provided suggestions to further clarify the wording of the assessment question stems and answer choices.

### Data analysis

Consensus was evaluated not only as observed, but also relative to the degree of consensus that would be expected by chance, using a content validity ratio^39^. Lawshe’s content validity ratio (CVR) method was developed to determine the validity of panellists’ assessments of essential knowledge and skills. It is a linear transformation of the percent consensus, such that a higher degree of consensus is weighted more heavily^39^. This transformation increases the confidence that the consensus level observed reflects the true level of agreement, and allows for the elimination of items that ‘might reasonably have occurred through chance’^39^.

For a sample of this size (*N* = 30), the critical value for which the degree of consensus exceeds that which would be predicted by chance was 0.33^40^. Items with a CVR greater than 0.33 were retained and processed for Round 2. Items with a CVR between 0 and 0.33 were re-posed to the group. Items with a CVR below 0 were excluded from further rounds.

For items for which consensus was not achieved in Round 1 (e.g. CVR was between 0 and 0.33), the degree of consensus overall and for each subgroup was shown to participants, along with the question in Round 2. Panellists were asked to re-evaluate the importance of the item, in light of their own views and the responses of other respondents.

In Rounds 2 and 3, analysis was primarily qualitative. Questions and answer stems were reformulated in response to panellists’ feedback using summative content analysis^41^. Both the frequency and salience of suggested changes were taken into account when making alterations to the items.

### Reporting Summary

Further information on research design is available in the Nature Research Reporting Summary linked to this article.

## Results

A total of 30 participants (out of 63 who were invited by email) contributed to Round 1. Participants were evenly distributed between the stakeholder groups, with ten patients, ten clinicians and ten policymakers responding. Twenty-six participants (86.7%) responded in Round 2 and 25 (83.3%) participated in Round 3.

### Quantitative analysis

Round 1 of the Delphi demonstrated an early degree of consensus on content domains. Of the 46 initial knowledge and skill items, 21 had a content validity ratio (CVR) over 0.33, meaning that agreement on the inclusion of that item was greater than predicted by chance. Nineteen items had a CVR below 0, indicating the item was irrelevant and should be excluded.

Table 1 shows responses based on the question topic. Approximately half of the questions in each content domain were retained. Bronchiectasis-specific knowledge items and communication skills questions were more likely to be included in subsequent rounds.

**Table 1:** Summary of Round 1 results.

Domain	Number of statements in domain	Statements where consensus was achieved (*n*)^a^
Knowledge	25	12
General	5	3
Bronchiectasis	15	8
Health Literacy	5	1
Skills	21	9
Daily Habits	6	3
Response to events	7	2
Communication	8	4

^a^Consensus was defined as a content validity ratio (CVR) greater than or equal to 0.33. CVRs range in value from −1 to 1. When greater than 50 percent of participants agree, the CVR is 0. The more conservative CVR ≥ 0.33 accounts for agreement greater than would be predicted by random chance.

In Round 2, sample questions and answers for the assessment were posed. One item had a CVR below 0 and was dropped, leaving 20 items in the scale. Because of attrition in the response rate, the CVR threshold used in Round 2 was 0.39 instead of 0.33. This means that at least 18 of the 26 respondents in this round needed to rank an item as essential for it to be included.

In Round 3, participants reviewed the changes made to the scale and affirmed the inclusion or exclusion of items. Again because of attrition, the CVR threshold was raised to 0.44. On average the degree of consensus in Round 3 was 97.2%, with unanimous inclusion of 13 items.

### Qualitative analysis

Modifications were made to question stems and answer choices in response to qualitative feedback. Feedback came in five general forms:

1. The question did not test the concept as intended.
2. The item referred to local terminology or processes that would not be understood by all respondents.
3. The wording or terminology was confusing, generally.
4. The item concerned non-essential information that patients shouldn’t be expected to know outright, but rather should have the skills to find when needed.
5. The ‘right’ answer would vary by patient, and thus answers could not be standardised.

Modifications were chosen based on suggestions made by panellists. The feedback was quite detailed, and in certain instances suggestions were adopted wholesale. In others, comments from several respondents were combined to form new questions or answer choices. Special attention was paid to items to which multiple panellists responded similarly.

### Final assessment tool

The output of this process was a 20-item assessment tool to measure patients’ self-management capabilities. The questions took various forms: simple multiple choice (*n* = 5), true/false (*n* = 4), ‘check all that apply’ (*n* = 3) and situational vignettes (*n* = 8). Each assessment item addressed either a piece of knowledge or skill that is necessary to self-manage effectively and safely. The purpose of the skill-based vignettes was to give patients the opportunity to express how they would handle given situations, showing that they can apply the knowledge that they have.

The assessment contains two overarching domains: knowledge and skills. The knowledge domain consists of two sub-domains: general health knowledge and bronchiectasis-specific knowledge. Within those, general health knowledge is based in general health literacy, and especially in understanding the importance of smoking cessation. Bronchiectasis-specific knowledge addresses information about disease characteristics and medication use. The skills domain consists of three sub-domains: symptom management, communication and addressing deterioration. Symptom management consists of general healthy habits and airway clearance exercises. Communication concerns the ability to assert preferences and raise concerns with healthcare professionals. Addressing deterioration tests the ability to recognise a potential infection and select the most appropriate level of care for a given change in symptoms.

Psychometric best practice advocates at least two items per content domain^19^. Table 2 presents a content validity matrix for the final assessment items.

**Table 2:** Content validity matrix for the assessment tool.

	Knowledge				Skills					
	General health knowledge		Bronchiectasis-specific knowledge		Symptom management		Communication		Addressing deterioration	
Item	Health literacy	Smoking cessation	Disease characteristics	Medications	Healthy habits	Airway clearance	Assert preferences	Raise concerns	Identify possible infection	Seek appropriate level of care
1. Sources of health information	x									
2. Name of disease			x							
3. Basic pathophysiology			x							
4. Staying hydrated					x					
5. Healthy diet					x					
6. Antibiotic resistance	x									
7. Smoking and lung damage		x								
8. Second-hand smoke		x								
9. Airway clearance, frequency						x				
10. Airway clearance, duration						x				
11. Asserting treatment preferences							x			
12. Watchful waiting									x	x
13. Starting a rescue pack									x	x
14. Recognising an emergency									x	x
15. Watchful waiting									x	x
16. Special considerations for bronchiectasis				x				x		
17. Raising concerns about medications				x				x		
18. Advocating for needed care							x			
19. Regular medications				x						
20. First-line antibiotics				x						

The full assessment tool is presented in Supplementary Note 2. Anticipated correct answers are shown in bold.

## Discussion

This is the first and only tool to assess the knowledge and skills patients need to self-manage bronchiectasis, a disease for which self-management is critical, but formal self-management support is lacking. The study leveraged the expertise of 30 people in three stakeholder groups (bronchiectasis patients, clinicians and policymakers) to develop the Self-Management Abilities Test.

Bronchiectasis is a relatively rare disease for which there are few disease-specific self-management programmes^16^, but significant information needs^17^. Whilst previous measures like the Lung Information Needs Questionnaire to ask patients for their perceptions of the adequacy of information they receive from clinicians^25^, it does not address patients’ ability to recall or act upon that information. The new Self-Management Abilities Test addresses this gap.

The study was conducted as a modified Delphi over three rounds. This iterative process allowed participants to provide increasingly granular insights and feedback. The assessment has the added advantage of having objectively correct answers, rather than being scaled psychometrically, for instance by rating on a Likert scale^19^.

This improves the ease of interpretation of results and their relevance to the clinical context. Patients’ responses to specific questions can be used as a starting point for patient education, discussions of self-management practices and more abstract concepts like perceived ability or self-efficacy for disease management. The assessment can also provide confidence that patients have a baseline level of knowledge and skills to assume self-management responsibilities, especially soon after diagnosis.

Rather than a self-management score or ranking, this assessment is intended to identify specific gaps in patients’ understanding and abilities, so that education efforts can be tailored to the specific needs of a given patient. Targeting deficits in this way can lead to more meaningful interactions between patients and healthcare professionals.

Participation was limited to the UK, which may limit generalisability for an international audience. An overrepresentation of viewpoints from North West London could have biased the questionnaire toward practices common to that region. When panellists surfaced concerns about processes or terminology, items were revised to apply more generally. Future work is needed to understand the applicability of this tool to other contexts.

Because bronchiectasis is an uncommon condition, identifying non-clinician policymakers with enough knowledge of the disease to contribute was challenging. This may have blurred the distinction between responses from clinicians and policymakers. On the other hand, the high degree of consensus across all stakeholders makes such subgroup analyses less relevant.

Certain topics that were judged to be important for self-management (e.g. understanding one’s own baseline symptoms) are simply not suited for assessments of this type. However, this assessment is intended as an indicator of a patient’s self-management ability, not a comprehensive measure of their knowledge and skills. Using this tool in combination with other indicators can give a fuller picture of a person’s self-management ability.

The self-management expertise of patients with chronic diseases is a patient safety issue. In some sense, patients are self-managing any time they make decisions or take actions related to their symptoms or disease. In the context of bronchiectasis, the daily process of managing symptoms, maintaining exercise and medication regimens, and recognising signs of exacerbation or deterioration, offer ample opportunities to improve the alignment of self-management decisions with evidence-based practice. It is important to know whether patients are equipped to self-manage in line with evidence-based guidance, in order to know how best to support them in that effort. It is imperative for the health system to ensure that patients have the capabilities to self-manage safely, either through self-management education, wraparound services, or other supports.

This assessment tool can be used as an indicator of the self-management ability—and therefore the resource needs—of people with bronchiectasis. In this way, it could guide the formulation of individual self-management plans in the context of a clinical encounter or annual review.

The Self-Management Abilities Test could also be used in the development and evaluation of self-management programmes for bronchiectasis. In addition to outlining the essential content of such a programme, the assessment itself could be used at baseline and at a follow-up to measure the attainment of learning outcomes. The assessment could be used in conjunction with the PRISMS taxonomy^37^ to clarify the roles of clinicians and patients in self-management interventions and to tailor those interventions to individuals.

Finally, providing patients with an inventory of what they know and are able to do relative to their disease can improve their confidence in their ability to self-manage. Likewise, patients will be able to demonstrate their capabilities to clinicians who may or may not be part of their regular care team. Clarity around what they are expected to know, and where they may need more support, can empower patients in communicating with healthcare professionals.

The assessment will undergo validity and reliability testing on a large sample. We will be testing whether the assessment produces similar results over time, whether it accurately describes the concepts we are interested in measuring, and whether there is an association between a person’s self-management ability and other characteristics.

The rationale for developing this assessment was to provide both clinicians and patients with a tool to facilitate discussions about patients’ preparedness to self-manage bronchiectasis (and to do so safely). However, it will be important to evaluate the extent to which the tool is useful in practice. A process evaluation, gathering the perspectives of patients and clinicians on using the tool, will accompany the validation study.

The Self-Management Abilities Test in its current form is specific to bronchiectasis, but many of the items are general health knowledge and skills that may apply to other chronic diseases. Possible extensions of this work include producing a generic short form of the assessment that can be used for multiple conditions or to replicate this method for other disease areas.

Self-managing a chronic disease requires specialised knowledge and skills, but self-management ability for bronchiectasis has not been well articulated to date. This study established that:

1. There is broad consensus among patients, clinicians and policymakers around the knowledge and skills required for effective self-management of bronchiectasis, and
2. It is possible to measure those key knowledge and skills with a standard assessment tool.

Identifying individuals’ strengths and needs in these areas will allow for more targeted provision of services and supports, more appropriate opportunities for patient engagement and more equitable and efficient use of resources.

## Supplementary information

Supplementary Information

Reporting Summary

## Data Availability

The datasets generated and analysed during the current study are available from the corresponding author on reasonable request.
