# Supplementary material for: The self-management abilities test (SMAT): a tool to identify the self-management abilities of adults with bronchiectasis
Source: NPJ Prim Care Respir Med. 2022 Jan 14;32:3. doi: 10.1038/s41533-021-00265-5 (PMC8760272; doi:10.1038/s41533-021-00265-5)
Supplement: Supplementary file 1 — Supplementary Information [file 41533_2021_265_MOESM1_ESM.pdf]

SUPPLEMENTARY INFORMATION

Supplementary Note 1: Initial 46 Items Posed in Round 1 Questionnaire.

For each of the following questions, please rate your level of agreement that the item is an appropriate measure of the knowledge, skills, or attitudes of patients as relates to self-management of bronchiectasis.

[Response scale: 7-point Likert; 1=essential, 7=not relevant]

Knowledge

Health literacy (HL)

To what extent is it important for patients to know…

1. Where to find appropriate and accurate information about their health?
2. How to recognize, pronounce, and use common medical terms?
3. How to use basic arithmetic?
4. The meanings of simple proportions and fractions?
5. The general concepts of risk and uncertainty?

Bronchiectasis-specific information (BSI)

To what extent is it important for patients to know…

1. The name of their disease?
2. The pathophysiology of their disease?
3. The names of medications they take on a regular basis?
4. The dosage of medications they take on a regular basis?
5. How their regularly prescribed medications work?
6. How to describe their baseline symptoms?
7. The contact information of a practice nurse or other professional?
8. The name of the organism(s) with which they are colonised?
9. The names of the first-line antibiotics they take in the event of infection/exacerbation?
10. The length of the course of antibiotics they should take?
11. How bronchiectasis differs from other respiratory diseases?
12. Other vocabulary (e.g. scarring) that may be used to describe bronchiectasis?
13. How their treatment will differ from patients with COPD (only)?
14. How to distinguish between flare-ups and exacerbations?
15. How bronchiectasis may interact with other diseases they have?

General health knowledge (GHK)

To what extent is it important for patients to know…

1. The connection between diet and exercise and improved health?
2. How antibiotics work?
3. The risks of overusing antibiotics?
4. Why smoking cessation is important?
5. The meaning of a positive versus negative test result?

Skills

Daily Habits (DH)

To what extent is it important for patients to…

1. Exercise or engage in physical activity daily?
2. Use chest-clearing techniques daily?
3. Take temperature daily?
4. Record symptoms daily?
5. Recognize and avoid triggers?
6. Use home oxygen or other breathing aids?

Response to events (RE)

To what extent is it important for patients to be able to…

1. Distinguish between symptoms that can be managed at home, with the help of a community pharmacist or GP, or require a trip to hospital?
2. Produce sputum cultures at home, without assistance of a physiotherapist?
3. Describe the importance of regular sputum culturing?
4. Interpret test results: e.g. sputum culture, bloods, spirometry, etc. ?
5. Recognise a first instance of pseudomonas in sputum culture?
6. Flag inappropriate antibiotic prescriptions (e.g. wrong drug, wrong length of prescription)?
7. Assess when to see GP vs specialist?

Communication (CS)

To what extent is it important for patients to…

1. Assert their preferences in a clinical encounter?
2. Communicate that their priorities may affect their ability/willingness to adhere to medical advice?
3. Raise concerns with a healthcare provider even when unsolicited?
4. Use clinically appropriate terms to describe their illness?

5. Follow up regarding discharge notes or other clinical communications?
6. Participate in a support group?
7. Engage in shared decision-making?
8. Take notes during medical appointments?

Attitudes

To what extent is it important for patients to report…

1. Confidence to manage pain?
2. Confidence to manage symptoms?
3. Confidence to address emotional distress, anxiety, or depression associated with the condition?
4. Confidence to seek help when necessary?
5. To understand their illness in the context of the rest of their lives?
6. A positive attitude with respect to their illness?
7. Confidence in the ability to cope with their illness?
8. Constructive attitudes and approaches to coping and viewing illness in perspective (heiQ)
9. Wanting to take an active role in their care?
10. Believing that they are ultimately responsible for managing their health condition?
11. Feeling positive about life in spite of their condition?
12. Feeling in control of their health and life?
13. Feeling adequately supported, socially and emotionally?

Supplementary Note 2: Self-Management Abilities Test (SMAT) – Bronchiectasis

We are interested in what you know about your lung condition, and the kinds of things you do to manage it. We don't expect you to know the answer to every question, so please just answer to the best of your ability.

1. There are lots of sources of health information - especially on the internet - but not all of them can be trusted. *Please circle all of the following* that you believe would be good sources of information about your long-term disease:

**Pharmacist**	**British Lung Foundation website**	Wikipedia	**GP surgery**
**Lung support group**	Social media	**NHS websites**	I don't know

2. What is the name of your long-term respiratory condition?

Bronchitis	**Bronchiectasis**	Bronchodilator	I don't know

3. Which of the following best describes the problem with your lungs?

I have high blood pressure in the blood vessels that carry blood to my lungs.
**My airways become widened and cannot clear themselves, so they get congested with thick mucus or sputum.**
I have a serious infection caused by inhaling water droplets from a contaminated water source.
I don't know.

4. Choose whether the following statement is True or False:

Staying hydrated helps keep the mucus in your lungs moving.

**True**	False	I don't know

5. Choose whether the following statement is True or False:

Eating a well-balanced diet may help manage your symptoms.

**True**	False	I don't know

6. Which of the following best describes why you should not overuse antibiotics?

Antibiotics are addictive, and you could develop a dependency.
**Bacteria can develop resistance to antibiotics, making them less effective.**
Viruses can grow stronger after being exposed to antibiotics.
Antibiotics are expensive.

7. Choose whether the following statement is True or False:

If a person quits smoking, it will undo the damage that has been done to their lungs.

True	**False**	I don’t know

8. Choose whether the following statement is True or False:

If a person smokes, the second-hand smoke is a risk to their loved ones’ health, too.

**True**	False	I don’t know

9. How often do you use chest-clearing techniques?

**About once a day, or more if I need it**	About once a week
About once a month	Rarely or never

10. How long should an airway clearance session last, generally speaking?

About 5 minutes	**About 20 minutes**	About 1 hour	About 2 hours

11. Which of the following best describes what you would do if your doctor recommended a treatment regimen that you didn’t like?

I would follow it to the best of my ability – the doctor knows best.
I would accept the advice, but not worry too much about following it.
**I would explain why I didn’t think the treatment was suitable for me.**
I would go to see a different doctor.

12. Michael is producing more sputum (mucus) than normal. He has no other change in his symptoms. What should he do?

**He should increase his airway clearance activities and stay hydrated, but not change his medication routine yet. He should continue to monitor his symptoms.**
He needs antibiotics. If he does not have a rescue pack that he can start straightaway, he should call his GP or consultant.
He should go to hospital immediately.
I don’t know.

13. Elisa has been feeling unwell for several days. She has been coughing more, feels breathless, and has stickier mucus. What should she do?

She should increase her airway clearance activities and stay hydrated, but not change her medication routine yet. She should continue to monitor her symptoms.
**She needs antibiotics. If she does not have a rescue pack that she can start straightaway, she should call her GP or consultant.**
She should go to hospital immediately.

I don’t know.

14. Tom has been feeling very breathless. He has noticed a reddish colour in his sputum. He also has a temperature and feels hot to the touch. What should he do?

He should increase his airway clearance activities and stay hydrated, but not change his medication routine yet. He should continue to monitor his symptoms.
He needs antibiotics. If he does not have a rescue pack that he can start straightaway, he should call his GP or consultant.
**He should go to hospital immediately.**
I don’t know.

15. Susan has noticed that her sputum colour has changed, and she is a bit more tired than normal. What should she do?

**She should increase her airway clearance activities and stay hydrated, but not change her medication routine yet. She should continue to monitor her symptoms.**
She needs antibiotics. If she does not have a rescue pack that she can start straightaway, she should call her GP or consultant.
She should go to hospital immediately.
I don’t know.

16. Julie believes that she is coming down with a chest infection. Her GP prescribes a 5-day course of amoxicillin 500mg, 3 times a day. Based on previous exacerbations she’s experienced, she doesn’t believe this is right.

What might be incorrect about this prescription for Julie?

**Julie should be on a longer course of antibiotics – at least 14 days.**
Julie should be taking the antibiotic more frequently – 5 times a day is recommended.
This prescription looks right to me.
I don’t know.

17. Julie believes that she is coming down with a chest infection. Her GP prescribes a 5-day course of amoxicillin 500mg, 3 times a day. Based on previous exacerbations she’s experienced, she doesn’t believe this is right.

What would you advise Julie to do in this situation?

Julie should take this medication as prescribed.
Julie should file a complaint with PALS.
**Julie should remind the GP of her chronic condition, and ask if there are specific prescribing guidelines for her.**
Julie should register with a different GP.

18. Over the past few days, your symptoms have become noticeably worse. It's not an emergency, but you do not want it to become one. What do you do?

My GP practice is very busy, and I don't want to be a bother. I will just ride this out.
I make an appointment through the normal channels; no need to jump the queue.
**I call the practice, and explain that my symptoms mean I need a same-day appointment.**
I go to A&E. Better safe than sorry.

19. Do you take any of the following medications for your lung condition? *Please circle all that apply, or write in your own answer.*

Aminophylline	Azithromycin 250mg daily	Azithromycin 250mg three times a week
Carbocisteine 750mg three times daily	Colomycin nebuliser	Doxycyline long term
Gentamycin nebuliser	Hypertonic saline twice daily	7% saline 4mL TDS
0.9% saline nebuliser	ICS and combination inhaler	Macrolide antibiotic
Montelukast	Mucolytic	Nasal steroid
Physiotherapy device - Acapella / Flutter etc	Prednisolone	SABA inhaler
Salbutamol inhaler 100mcg as required	Ventolin inhaler	I don't know
I don't take any medications regularly for my lung condition	I take medications regularly for my lung condition, but I don't know what they are called	I take medications regularly for my lung condition, but they are not on this list: _________________

20. Which of the following is the antibiotic you usually take in the event of an exacerbation of your lung condition? *Please circle all that apply, or write in your own answer.*

Amoxycillin 1g, 3 times a day	Amoxycillin 500mg, 3 times a day
Augmentin (co-amoxiclav) 625mg, 3 times a day	Co-amoxiclav plus amoxycillin, 3 times a day
Doxycycline 200mg once a day	Doxycycline 200mg first then 100mg daily
Clarithromycin 500mg twice a day	Ciprofloxacin 500mg twice a day
Ciprofloxacin 750mg twice a day	Septrin 960mg twice a day
I don't know	I don't take any antibiotics for my lung condition
I take antibiotics for my lung condition, but I don't know what they are called	I take antibiotics for my lung condition, but they are not on this list: _________________
