# Supplementary material for: The self-management abilities test (SMAT): a tool to identify the self-management abilities of adults with bronchiectasis
Source: NPJ Prim Care Respir Med. 2022 Jan 14;32:3. doi: 10.1038/s41533-021-00265-5 (PMC8760272; doi:10.1038/s41533-021-00265-5)
Supplement: Supplementary file 2 — Reporting Summary [file 41533_2021_265_MOESM2_ESM.pdf]

nature portfolio

Corresponding author(s): Katelyn R Smalley

Last updated by author(s): Sep 2, 2021

Reporting Summary

Nature Portfolio wishes to improve the reproducibility of the work that we publish. This form provides structure for consistency and transparency in reporting. For further information on Nature Portfolio policies, see our Editorial Policies and the Editorial Policy Checklist.

Statistics

For all statistical analyses, confirm that the following items are present in the figure legend, table legend, main text, or Methods section.

n/a	Confirmed	
☐	☒	The exact sample size (*n*) for each experimental group/condition, given as a discrete number and unit of measurement
☐	☒	A statement on whether measurements were taken from distinct samples or whether the same sample was measured repeatedly
☒	☐	The statistical test(s) used AND whether they are one- or two-sided<br>*Only common tests should be described solely by name; describe more complex techniques in the Methods section.*
☒	☐	A description of all covariates tested
☒	☐	A description of any assumptions or corrections, such as tests of normality and adjustment for multiple comparisons
☒	☐	A full description of the statistical parameters including central tendency (e.g. means) or other basic estimates (e.g. regression coefficient) AND variation (e.g. standard deviation) or associated estimates of uncertainty (e.g. confidence intervals)
☒	☐	For null hypothesis testing, the test statistic (e.g. *F*, *t*, *r*) with confidence intervals, effect sizes, degrees of freedom and *P* value noted<br>*Give P values as exact values whenever suitable.*
☒	☐	For Bayesian analysis, information on the choice of priors and Markov chain Monte Carlo settings
☒	☐	For hierarchical and complex designs, identification of the appropriate level for tests and full reporting of outcomes
☒	☐	Estimates of effect sizes (e.g. Cohen's *d*, Pearson's *r*), indicating how they were calculated

Our web collection on statistics for biologists contains articles on many of the points above.

Software and code

Policy information about availability of computer code

Data collection	The questionnaires were administered online via Qualtrics software licensed to Imperial College London.
Data analysis	Data was stored and managed in Excel, and quantitative analyses were completed either in R or Excel.

For manuscripts utilizing custom algorithms or software that are central to the research but not yet described in published literature, software must be made available to editors and reviewers. We strongly encourage code deposition in a community repository (e.g. GitHub). See the Nature Portfolio guidelines for submitting code & software for further information.

Data

Policy information about availability of data

All manuscripts must include a data availability statement. This statement should provide the following information, where applicable:

- Accession codes, unique identifiers, or web links for publicly available datasets
- A description of any restrictions on data availability
- For clinical datasets or third party data, please ensure that the statement adheres to our policy

The datasets generated and analysed during the current study are available from the corresponding author on reasonable request.

Field-specific reporting

Please select the one below that is the best fit for your research. If you are not sure, read the appropriate sections before making your selection.

☐ Life sciences ☒ Behavioural & social sciences ☐ Ecological, evolutionary & environmental sciences

For a reference copy of the document with all sections, see nature.com/documents/nr-reporting-summary-flat.pdf

Behavioural & social sciences study design

All studies must disclose on these points even when the disclosure is negative.

Study description	This study employed an online, modified Delphi method. It took place over the course of three rounds with the same participants. The study generated both qualitative and quantitative data.
Research sample	This study, which took place in the UK, solicited expert opinions from three stakeholder groups:<br>1. Adult patients with bronchiectasis (either with or without cystic fibrosis)<br>2. Clinicians (general practitioners, respiratory consultants/specialists, respiratory physiotherapists, specialist nurses, pharmacists) who currently work in a clinical capacity, and who have experience treating bronchiectasis<br>3. Policymakers with experience of bronchiectasis (broadly conceived as people whose current responsibilities include not only direct patient care, but also the development of policies and procedures for the care of these patients either at the multi-disciplinary team, trust, regional, or national level)<br>This range of stakeholders were chosen to provide a multi-faceted view of self-management ability.
Sampling strategy	Reflecting sample size guidance for Delphi studies, ten participants represented each stakeholder group, for a panel of 30 participants in total (de Villiers, de Villiers, and Kent 2005).<br>Patients were recruited online via the Voice Global and People in Research networks of lay researchers, Twitter, the British Lung Foundation's Breathe Easy support groups, and through informal networks of lay representatives at Imperial College. Clinicians were recruited via respiratory professional societies such as BronchUK and the British Thoracic Society, publicly available profiles, and personal references. Policymakers were identified via public research and policy profiles, and leadership of professional societies, research groups, or major clinical studies of bronchiectasis. The panel was recruited from across the UK.
Data collection	Data was collected via online questionnaires administered through Qualtrics. No one aside from the participant was present during data collection. No other data collection instruments were used.
Timing	Round 1: 14 Jun 2019-24 Sep 2019<br>Round 2: 26 Nov 2019-10 Jan 2020<br>Round 3: 26 Feb 2020-23 Apr 2020
Data exclusions	No data were excluded from analysis.
Non-participation	A total of 30 participants contributed to Round 1. Twenty-six participants (86.7%) responded in Round 2, and 25 (83.3%) participated in Round 3.
Randomization	Participants were not randomized.

Reporting for specific materials, systems and methods

We require information from authors about some types of materials, experimental systems and methods used in many studies. Here, indicate whether each material, system or method listed is relevant to your study. If you are not sure if a list item applies to your research, read the appropriate section before selecting a response.

Materials & experimental systems

n/a	Involved in the study	
☒	☐	Antibodies
☒	☐	Eukaryotic cell lines
☒	☐	Palaeontology and archaeology
☒	☐	Animals and other organisms
☐	☒	Human research participants
☒	☐	Clinical data
☒	☐	Dual use research of concern

Methods

n/a	Involved in the study	
☒	☐	ChIP-seq
☒	☐	Flow cytometry
☒	☐	MRI-based neuroimaging

Human research participants

Policy information about studies involving human research participants

Population characteristics	See above
Recruitment	Patients were recruited online via the Voice Global and People in Research networks of lay researchers, Twitter, the British Lung Foundation's Breathe Easy support groups, and through informal networks of lay representatives at Imperial College. Clinicians were recruited via respiratory professional societies such as BronchUK and the British Thoracic Society, publicly available profiles, and personal references. Policymakers were identified via public research and policy profiles, and leadership of professional societies, research groups, or major clinical studies of bronchiectasis.<br><br>Participation was limited to the UK, which may limit generalisability for an international audience. An overrepresentation of viewpoints from North West London could have biased the questionnaire toward practices common to that region. When panellists surfaced concerns about processes or terminology, items were revised to apply more generally. Future work is needed to understand the applicability of this tool to other contexts.<br>Because bronchiectasis is an uncommon condition, identifying non-clinician policymakers with enough knowledge of the disease to contribute was challenging. This may have blurred the distinction between responses from clinicians and policymakers. On the other hand, the high degree of consensus across all stakeholders makes such sub-group analyses less relevant.
Ethics oversight	UK NHS Health Research Authority

Note that full information on the approval of the study protocol must also be provided in the manuscript.
